# A scoping review of nutrition education interventions to improve competencies, lifestyle and dietary habits of medical students and residents

**DOI:** 10.1017/jns.2023.16

**Published:** 2023-03-02

**Authors:** Victor Mogre, Bright Yammaha Amoore, Patience Kanyiri Gaa

**Affiliations:** 1 Department of Health Professions Education and Innovative Learning, School of Medicine, University for Development Studies, Tamale, Ghana; 2 Department of Nutritional Sciences, School of Allied Health Sciences, University for Development Studies, Tamale, Ghana

**Keywords:** Competencies, Dietary habits, Lifestyle behaviours, Medical students, Nutrition education

## Abstract

We reviewed the available research and gave an overview of the effects of nutrition education interventions (NEIs) on medical students’ and residents’ knowledge of nutrition, attitudes towards nutrition care, self-efficacy, dietary practices and readiness to offer nutrition care. From 28 May through 29 June 2021, we searched Google Scholar, PubMed, ProQuest, Cochrane and ProQuest to retrieve 1807 articles. After conducting de-duplication and applying the eligibility criteria and reviewing the title and abstract, 23 papers were included. The data were descriptively and narratively synthesised, and the results were displayed as frequencies, tables and figures. Twenty-one interventions were designed to increase participants’ knowledge of nutrition-related topics, and eighteen studies found that nutrition knowledge had significantly improved post-intervention. Only four of the eleven studies that reported on attitudes about nutrition post-intervention showed a meaningful improvement. The self-efficacy of participants was examined in more than half of the included studies (*n* 13, 56⋅5 %), and eleven of these studies found a significant increase in the participants’ level of self-efficacy to offer nutrition care post-intervention. At the post-intervention point, seven interventions found that dietary and lifestyle habits had significantly improved. The review demonstrated the potential of NEIs to enhance participants’ dietary habits and nutrition-related knowledge, attitudes and self-efficacy. Reduced nutrition knowledge, attitude and self-efficacy scores during the follow-up, point to the need for more opportunities for medical students and residents to learn about nutrition after the intervention.

## Introduction

Morbidity and death in all age groups are considerably increased by poor diets. One in five fatalities worldwide are caused by poor nutrition. In 2017, 11 million deaths were attributable to dietary risk factors, with 3 million deaths each due to high salt intake and poor whole grain intake, and 2 million due to low fruit intake^(1)^.

Promoting healthy eating habits is a recognised strategy to lessen the impact of malnutrition. Since doctors are usually the gatekeepers in the healthcare system, they play an important role in encouraging people to eat healthy and live a healthy lifestyle. Doctors can assist individuals by giving nutrition counselling, nutrition assessments or identifying and recommending patients to seek professional dietetic care, all of which are referred to as nutrition care.

Doctors are typically seen as trustworthy sources of nutrition by the general public and are expected to provide nutrition care. The Behavioural Risk Factor Surveillance System study revealed a roughly threefold increase in patients’ attempts to treat their nutrition issues following physician advice^(2)^. Thus, it should come as no surprise that the majority of professional bodies in a number of countries list nutrition care as one of the responsibilities that doctors should take on when delivering healthcare^(3)^.

Nonetheless, physicians worldwide continue to underutilise this opportunity to render nutrition care to their clients. Doctors struggle to provide nutrition care due to a lack of training in nutrition counselling, limited expertise and low confidence^(3,4)^. The vast majority of practicing doctors, medical students and incoming interns frequently report being dissatisfied and unwilling to counsel patients with nutritional issues^(4,5)^.

Some nutrition education interventions (NEIs) have been carried out over the years to improve medical students’ nutrition education experiences, with results published in the literature, in order to promote effective nutrition care provision. However, there has been a scarcity of critical systematic reviews of the literature to help inform the design and development of these interventions on the competencies and dietary habits of medical students and residents. There have been three previous reviews of this phenomenon^(3,6,7)^. Mogre *et al.*^(6)^ conducted a realist review of the literature to determine what types of educational interventions work, how, for whom, why and under what conditions to improve doctors’ and other healthcare professionals’ competencies and nutrition care delivery. Crowley *et al.*^(3)^ conducted a critical review of the literature in 2019 and reported on the types of nutrition education provided to medical students, but did not report on their impact on medical students’ and residents’ competencies. Sunguya *et al.*^(7)^ reported on the effectiveness of in-service nutrition training programmes on health workers’ nutrition knowledge, counselling skills and child undernutrition management practices. Although the review by Mogre *et al.*^(6)^ reported on the impact of NEIs on medical students’ competencies, it did not extensively evaluate the impact of NEIs on medical students’ own dietary habits, only included studies published until 2014, and has not been updated since. Crowley *et al.*^(3)^ conducted a review that included both interventional and non-interventional studies, as well as studies published up to 2018.

This scoping review aims to map current literature and provide a summary of the impact of NEIs on nutrition care competencies, self-efficacy and dietary habits of medical students and residents as a prelude to conducting a comprehensive systematic review and meta-analysis of the literature. This will provide information that both academics and practitioners may find useful.

## Methods

The study followed the scoping review guidelines proposed by Arksey and O'Malley^(8)^ and the recommendations of Levac *et al*.^(9)^. We used these guidelines to screen data from existing databases and then reported in accordance with the PRISMA extension for scoping review^(10)^.

### Eligibility criteria

Studies were chosen based on the following eligibility criteria.

*Participants:* Medical students and residents.

*Outcomes:* Eligible studies included those that reported on nutrition knowledge, attitudes towards nutrition care, self-efficacy, nutrition counselling skills, dietary and lifestyle practices, and readiness to provide nutrition care. Intervention studies aimed at changing the dietary and lifestyle habits of medical students and residents were also considered.

*Study design:* Randomised control trials, quasi-experimental studies and prospective studies.

*Time frame:* From January 2010 to June 2021. Only published studies were considered.

*Language:* Only English-language publications were considered.

### Exclusion criteria

All systematic reviews and one-time studies were excluded. Interventions aimed at medical doctors or family physicians were also excluded from the study. Furthermore, commentaries, case studies, conference proceedings, non-peer-reviewed papers, opinion pieces, letters to the editor and abstracts were not accepted.

### Information sources and search strategy

We searched four electronic databases, including PubMed, ProQuest, Cochrane and Google Scholar, to find relevant literature (for grey literature). From 28 May 2021 to 29 June 2021, the literature was searched, and email alerts were set up to notify of newly published articles. BYA developed the search strategy based on the study's main research question, which was then reviewed by VM. Keywords and databases pertinent to the study were identified by BYA, VM and a librarian. Appropriate MESH terms as well as free text of keywords were identified and used as needed. Keywords included ‘nutrition care’, ‘nutrition care competency’, ‘knowledge’, ‘attitudes’, ‘nutrition counselling’, ‘nutrition education’ and ‘medical students’ (undergraduate students or trainees or future doctors). The appropriate Boolean operators were used. The search strategy was developed first for PubMed and then applied to the other databases.

### Selection of relevant studies

All search results were exported into EndNote X9 reference manager for de-duplication and selection. BYA and VM independently screened the titles and abstracts against the eligibility criteria. Full-text screening was used for articles with insufficient information in the title and abstract to justify their inclusion. BYA and VM discussed the results of the screening process with the rest of the review team, highlighting areas of disagreement that were resolved through discussion. The reference list of the selected articles was searched for additional relevant articles to include.

### Data extraction/charting

We extracted data on population (i.e. medical students or interns or residents or student doctor or trainee doctor), sample size, study design, focus or objective of the study, educational level of participants, methods of teaching and learning, type of intervention (e.g. workshop, curriculum design), duration of intervention, outcome of intervention and study location. Components of the data extraction form was based on previous research^(6,11)^. Each full text was reviewed using this format, and the data were extracted into an Excel spreadsheet. BYA extracted the data, and the results were discussed with VM and the other team members. We did not assess the methodological quality of the included studies because we were scoping the literature for a more comprehensive systematic review.

### Data analysis and synthesis

The data were analysed in Microsoft Excel 2013. Descriptive analysis was performed and presented in the form of frequencies, percentages and narrative format. Furthermore, we classified the included studies based on the type of outcomes reported, NEI and study designs.

### Patient and public involvement

Patients and/or the public were not involved in the design, conduct, reporting or dissemination plans for this study.

## Results

**Fig. 1.**
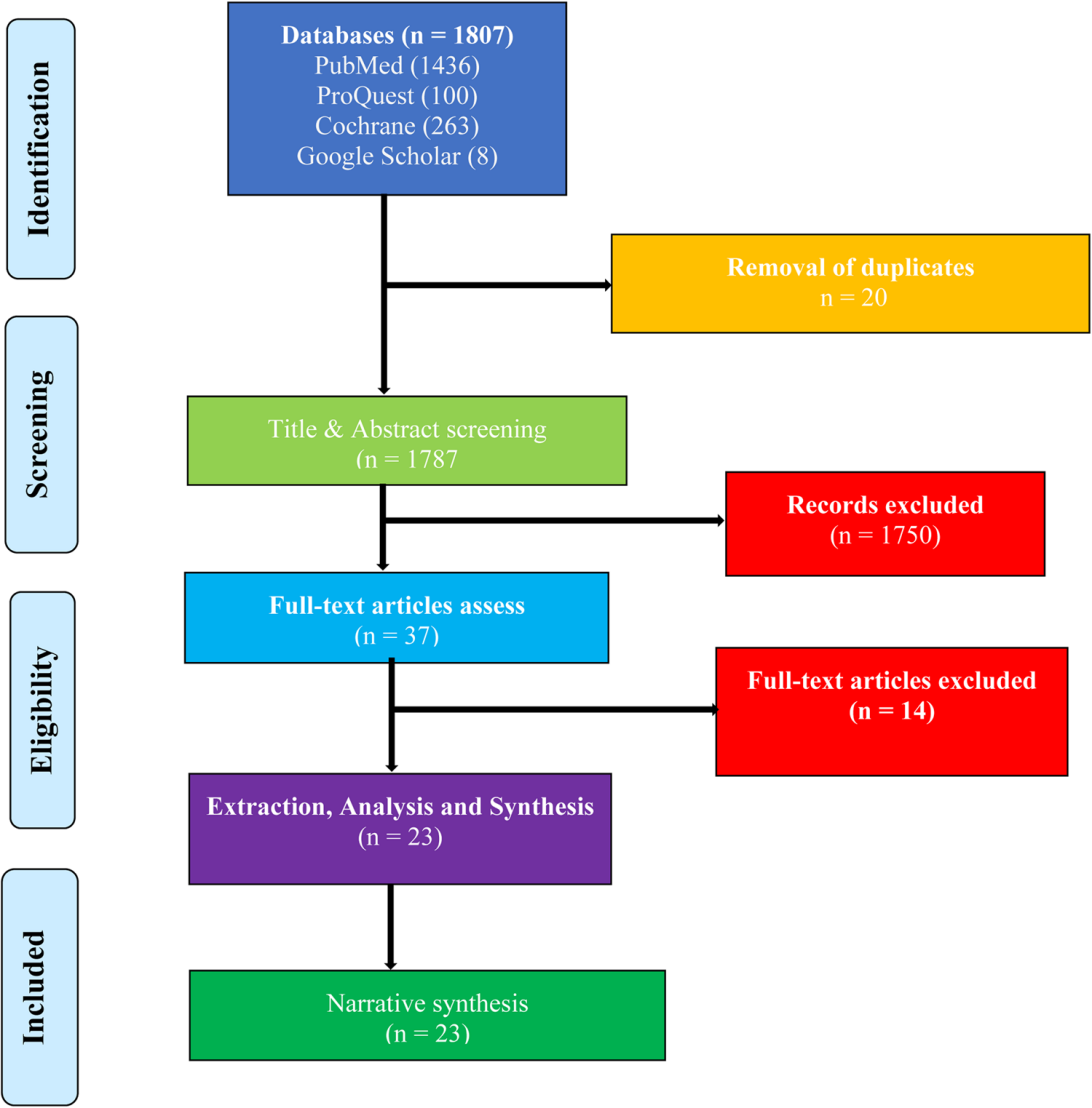
A PRISMA flowchart of the study selection process.

The four databases yielded 1807 articles. After de-duplication, there were 1787 articles left for screening. After removing 1750 studies based on title and abstract screening, the remaining 37 studies were subjected to full-text screening, with 23 articles found eligible and included in the analysis. The flowchart of the selection process is shown in Fig. 1.

Eighteen of the studies (78⋅3 %) were conducted in the United States of America (USA), while the remaining articles were conducted in the Netherlands, Indonesia, Iran, New Zealand and Portugal (shown in Table 1).

**Table 1.** Characteristics of the included studies (*n* 23)

Author (s) and year	Study location	Intervention objective(s)	Study design	Sample size and participants	Intervention type/teaching and learning methods	Duration	Outcomes
Rothmans *et al.*^(12)^	USA (Perelman School of Medicine)	Culinary medicine on the knowledge, confidence and personal dietary habits and confidence of counselling in medical students.	Pre- and post-course surveys without control group	31 fourth-year medical students	Nutrition instructions Demonstrations and practical sessions Lectures Case-based talks	8 weeks	Improved confidence of providing nutrition counselling for behaviour change. Insignificant change in dietary habits except for fruit consumption. Conscious efforts towards modification of food behaviours
Carnes *et al.*^(13)^	USA (University of Connecticut, School of Medicine)	Equip medical trainees with fundamental nutrition guidelines and skills for dietary history taking and counselling	Pre–post-survey and 2-months post-intervention survey with historical control	81 first-year medical students	1-h online lecture Standardised patient (SP) training 4 h on assessment of interviewing and interpersonal skills 2 h on case portrayal		Improvement in confidence of nutrition counselling among students.
Bertz *et al.*^(14)^	US (Boston School of Medicine)	Addressing common dietary behaviour patterns Providing evidence-based tools for dietary assessment and counselling.	Pre-test–post-test and a follow-up after 1 month without control	66 fourth-year medical students But 42 completed the survey. 30 had complete data for analysis	A single recurring seminar Interactive session Role-plays Monthly didactic sessions Lecture-based (PowerPoint presentations)	8 months	Significant increase in nutrition knowledge immediately after the intervention and 1-month post-intervention Significantly improved the confidence of accurately obtaining dietary history of clients Improvement in dietary counselling
Rubin *et al.*^(15)^	USA (Boston, Massachusetts)	Impact of a diabetes curriculum on the nutrition knowledge of internal medicine residents.	Pre- and 1-year post-curriculum survey without control group	91 internal medicine residents	Diabetic curriculum Lectures Case-based diabetes rotations	1 year	Significant increase in residents’ nutrition knowledge
Lang *et al.*^(16)^	USA (Remington neighbourhood of Baltimore)	Improving resident physician nutritional counselling and culinary skills	Pre–post without control group	16 second-year general preventive medicine residents	Three 4-h workshops Didactic PowerPoint presentations Interactive demonstrations	10 months	Significant improvement in the incidence of home-prepared foods
Pang *et al.*^(17)^	USA (Keck School of Medicine)	Teach students how to turn culinary skills into tools for counselling in other to boast their confidence in preparing healthy diets	Pre- and post-course survey without control	47 second-year medical students	90-min cooking session 30 min of communal eating and session debriefing 30-min discussion on how diseases and MyPlate relate to health	6 weeks	Class was well-received Improved students’ nutrition knowledge, confidence in lifestyle counselling, and personal culinary skills
Wood *et al.*^(18)^	USA (Wayne State University School of Medicine)	The efficacy of a hands-on culinary nutrition curriculum on medical students’ personal dietary habits and perceived preparedness to counsel patients on a healthy diet.	Pre–post and two (2) months post-test without control group	10 first-year medical students	Designed as a practical complement to a lecture-based Clinical Nutrition course 20 min of culinary theory didactic instruction 10 min of demonstrated culinary technique instruction 80 min of supervised cooking in small groups 10 min of an interactive nutrition discussion	1 month	Improved the level of preparedness to counsel patients on healthy diet both immediately and 2 months post-intervention. Significantly improved culinary knowledge immediately and 2 months post-intervention. Significantly improved dietary behaviour. Significantly improved self-rated attitude towards healthy dietary counselling.
Afaghi *et al.*^(19)^	Iran	Improve clinical skills towards the role of nutrition in chronic disease, metabolic, heart, renal and intestinal disease Assessment of current dietary intake and weight management.	Pre–post and one (1) month post-test without control group	46 fourth and fifth-year medical students	Case-based problem-oriented tutorial curriculum design	4 weeks	Significantly increased the nutrition knowledge score of students immediately post-intervention
Sjarif *et al.*^(20)^	Indonesia	Effectiveness of a new paediatric nutrition module	Quasi-experimental study without control group and 3-months retention test	53 fifth-year (5th year) medical students	Lectures Paper-based workshops Case practice (Role-play) Videos and interactive learning	7 months	Significantly improved students’ nutrition knowledge and counselling skills
Gayer *et al.*^(21)^	USA (California)	Comprehensive obesity curriculum on the knowledge and attitude of future doctors towards people with obesity	Pre- and post-curriculum survey with control group	718 first, second, third and fourth-year students	Lectures Interactive case study simulation Virtual patient case presentations Providing reading material	First year 2015; 5 h, first year 2016 through 2018; 6 h Second year 2015; 3 h	The intervention significantly increased the obesity-related knowledge of students Significant improved the attitude towards people with obesity
Schlair *et al.*^(22)^	USA (New York)	Impact of a nutrition programme on the nutritional knowledge, confidence of counselling and the impact of students’ behaviour on outcome.	Pre–post and 2 weeks post-intervention survey without control group	162 first-year medical Students	Lectures Interactive workshop	2 h	There was a significant increase in students’ nutrition knowledge and confidence in nutrition counselling. Significant improvement in students’ attitude towards nutrition. Improved students’ dietary habits e.g. plenty fruits and vegetables consumption and avoidance of fatty food intake.
Crowley *et al.*^(23)^	New Zealand (University of Auckland)	Impact of a nutrition intervention on the dietary habit and self-efficacy to improve personal behaviours and counselling.	Pre–post-course survey with control group	239 medical students and sixty-one (25⋅5 %) participated in baseline and post-intervention survey	Didactic learning Small interactive sessions	12 weeks (involving 13 contact hours)	Significantly improved students’ dietary habits e.g. wholegrain food intake Improved knowledge of dietary choices There was an insignificant increase in attitude towards dietary counselling. No significant improvement in confidence towards nutrition care to family and friends.
Coppoolse *et al.*^(24)^	Netherlands	Impact of nutrition education on the nutrition knowledge and intentions towards dietary counselling.	Baseline and post-intervention survey with control group	118 medical students	Lectures	10-weeks course 24-h course	Significantly improved the nutrition knowledge Improved intention of providing dietary counselling in medical students
Spear *et al.*^(25)^	USA (Methodist Hospital in Houston, Texas)	Pilot nutrition education programme in improving residents’ knowledge of nutrition needs in the critically ill clients.	Prospective observational pilot study with pre- and post-testing and 3-months post-testing without control group	8 post-graduate year-one surgery residents	Lectures Interactive case studies	12 months	Improved both short-term and long-term ICU nutrition knowledge of residents.
Nawaz *et al.*^(26)^	USA (Griffin Hospital)	New curriculum and rotation in lifestyle medicine for preventive medicine residents.	Baseline survey and follow-up survey with control group	20 preventive medicine residents	Clinical rotations Didactic lectures Demonstrate Interactive sessions Web-based modules Distance learning Educational Conferences	2 weeks	No significant change in attitude towards nutrition care. No significant change in nutrition and lifestyle knowledge. No significant change in confidence towards discussing nutrition and lifestyle with clients. No significant improvement in self-reported residents’ quality of dietary habit.
Walsh *et al.*^(27)^	USA (Children's Hospital Boston)	Impact of change in curriculum (dedicated to integrated) on the nutritional knowledge and attitude of medical student.	Quasi-experimental design (Pre–post-survey) without control group	Year-2; 131 PMN and 135 ICN (responses received from 66 to 59, respectively)	45-min lecture 90-min small group exercise Problem-based learning Debates Self-assessment exercises	14 weeks (28 contact hours)	No significant change in nutritional knowledge and attitude of medical students. Significant dissatisfaction with their nutrition education
Mota *et al.*^(28)^	Portugal	Impact of Nutrition and Metabolism (NM) curricular unit (CU) on the attitude, behaviours, knowledge, confidence on future practice and adherence to the Mediterranean diet (MD) of first-year medical students.	Pre- and post-testing without control group	310 first-year medical students	Problem-based learning method Clinical case presentations Discussion of scientific evidence	2 months (75 contact hours)	Increased the nutritional knowledge of students Improved dietary habit of reading nutrition label and avoiding meals with high level of sugar and fat Increased the confidence of dietary counselling of people with type 2 diabetes, persons with increased LDL and clients under treatment for obesity.
Magallanes *et al.*^(29)^	USA (UT Southwestern School of Medicine)	Impact of culinary medicine elective on students’ understanding of interprofessional engagement for the management of lifestyle diseases, knowledge of healthy diet and confidence of dietary counselling in medical students.	Pre- and post-course survey without control group	64 first-year medical students	Culinary medicine (CM) elective course (Interprofessional education, teaching, CM demonstrations, nutrition case discussions)	2 years (3-h monthly kitchen session)	Significantly increased the confidence of discussing nutrition with clients immediately post-intervention Significantly increased students’ knowledge of Mediterranean diet and the role of dieticians in the clinical care of clients.
Desalvo *et al.*^(30)^	USA	Diabetics management	Pre- and post-curriculum survey without control group	89 residents	Online interactive PowerPoint presentation Discussions Learner-initiated questions and answers Case presentations	8 weeks	Significantly reduced the error of nutrition-related diabetes management in residents
Lewis *et al.*^(31)^	USA	Effect of nutrition modules delivered online in interactive and non-interactive formats.	Pre- and post-test design without control group	322 paediatric trainees	Online nutrition training involving ten interactive and non-interactive models Case discussion and applied learning activities	8 months	Significantly increased the nutritional knowledge of paediatric trainees
Monlezun *et al.*^(32)^	USA	Impact of hands-on culinary medicine and the traditional medical curriculum on knowledge of nutrition and lifestyle and attitude towards nutrition care.	Prospective multisite cohort study design with control and treatment exposure group	3248 first- to fourth-year medical trainees involving twenty medical schools	Post-class problem-based learning Lectures Simulation Hands-on cooking sessions Pre-class video lecture	5 years	Significantly improved the nutrition knowledge and lifestyle of students Improved their dietary habits.
Monlezun *et al.*^(33)^	USA (New York)	Effect of simulation-based medical education with hands-on cooking nutrition elective *v*. the traditional medical curriculum	Longitudinal comparative effectiveness study	627 first- to fourth-year medical students	Elective in nutrition education Problem-based learning Quiz Video lectures Rotations Simulation Seminars.	28-h student elective in nutrition education. Twice annually	Significantly improved the dietary habit of medical students immediately post-intervention e.g. vegetable consumption. Significantly improved the attitude of students towards nutrition care Significantly improved the nutrition knowledge of participants towards nutrition care
Frates *et al.*^(34)^	USA	Impact of an interactive lifestyle presentation on the nutrition care knowledge and attitude towards the management of chronic diseases	Pre- and post-test survey without control group	First- and second-year medical student	Interactive presentation on lifestyle medicine	1 h	Improved the confidence of counselling clients on healthy lifestyle behaviours Improved attitude of students towards providing patients with healthy lifestyle education

Table 2 summarises the study designs, methodological approaches and the format of intervention of the included studies. The articles used a variety of study designs, with the majority (*n* 15, 65⋅2 %) using a quasi-experimental design. The included studies had a total sample size of 6621 participants (median = 81 participants; interquartile range (IQR): 25⋅5 and 252⋅5). The studies with integrated participation (a combination of preclinical and clinical year students) had the largest sample size (median = 718, IQR: 672⋅5 and 1983). Slightly more than three-quarters of the studies (*n* 18, 78⋅3 %), used only survey questionnaires and a few studies (*n* 5, 21⋅7 %) combined surveys and open-ended questions. The majority of the surveys (*n* 14, 60⋅9 %) were conducted using validated questionnaires adapted from previous research. Eighteen (78⋅3 %) of the studies used a quantitative method, while the remaining (*n* 5, 21⋅7 %) used mixed methods (quantitative and qualitative methods combined). Mixed method approaches were used in studies that reported on participants’ feedback about the intervention (*n* 5, 21⋅7 %). The interventions were delivered through a variety of methods, including problem-based learning sessions, demonstrations, interactive case presentations, interactive case studies, interactive workshops, paper-based workshops, debates, hands-on experience self-assessment, visual patient presentation, culinary sessions, case-based presentations, video lecture and rotations in lifestyle medicine.

**Table 2.** Study designs, format of intervention and data collection methods

Variable	Frequency (%)
Study design	
Experimental	1(4⋅3)
Pre-test and post-test quasi-experimental design with control	7(30⋅4)
Pre-test and post-test quasi-experimental design with no control	13(56⋅5)
Longitudinal observational study	2(8⋅7)
Methodological approach	
Mixed method	5(21⋅7)
Quantitative	18(78⋅3)
Format of Intervention	
Pilot study	4(17⋅4)
Online training	3(13⋅0)
Seminar	1(4⋅3)
Curriculum	8(34⋅8)
Workshop	3(13⋅0)
Culinary elective course	1(4⋅3)
Artificial intelligence machine learning	1(4⋅3)
Elective course	1(4⋅3)
Interactive presentation on lifestyle medicine	1(4⋅3)
Data collection methods	
Questionnaire/survey only	18(78⋅3)
Questionnaire/survey with other methods (interview)	5(21⋅7)
Students	
Undergraduate, preclinical	10(43⋅5)
Undergraduate, clinical	3(13⋅0)
Residents	5(21⋅7)

### Nutrition-related knowledge

Almost all the studies (*n* 21, 91⋅3 %) assessed changes in study participants’ nutrition-related knowledge^(12,14–28,31–35)^. Eighteen of these studies (85⋅7 %)^(12,14–25,28,31–33,35)^ found a significant positive change in knowledge immediately following intervention. Three studies (*n* 3, 14⋅3 %) reported no change in knowledge or improvement without specifying whether the change was statistically significant^(26,27,34)^. Nawaz *et al.* found a slight but insignificant increase in residents’ knowledge on various lifestyle components after following a lifestyle medicine curriculum that included didactics, distance learning, educational conferences and a newly developed lifestyle medicine rotation programme in a study of twenty preventive medicine residents^(26)^.

The qualitative feedback of study participants demonstrates that students learned about nutrition-related disorders, and how to encourage the adoption of healthy dietary practices^(12)^. Except for four studies^(14,18,20,25)^, which had a two-time measurement and a follow-up (or third time measurement) assessment post-intervention, all the studies had two-time measurements (i.e. baseline and post-intervention). Two of these studies reportedly conducted a 3-month follow-up assessment^(20,25)^. One study each, included 1-month^(14)^ and 2-month^(18)^ post-intervention follow-up measurements. Except for one study, all the studies that had follow-up assessments reported a decrease in the mean knowledge scores of the participants at follow-up compared with immediately post-intervention but higher than the baseline score. For example, Wood *et al.*^(18)^ discovered that first-year medical students’ baseline mean knowledge score was 5⋅3 out of a possible 10⋅0 points, which increased significantly (*P* = 0⋅001) to 8⋅8 points immediately following the intervention and declined to 6⋅9 points 2 months later^(18)^. In addition, Berz *et al.*^(14)^ discovered a baseline score of 4⋅3 that significantly increased to 5⋅8 immediately post-intervention but declined to 5⋅2 at the 1-month follow-up assessment. In an observational prospective study, Spear *et al.*^(25)^ implemented nutrition education among surgery residents, with mean knowledge scores increasing from 45 % at baseline to 82 % immediately post-intervention and declining by 65 % 3 months later. Sjarif *et al.*^(20)^ found a knowledge score of 23⋅7 % at baseline that increased to 61⋅7 % post-intervention and 63 % at 3-months post-intervention using a quasi-experimental design.

### Attitude towards nutrition care

Of the twenty-three studies, 47⋅8 % (*n* 11) investigated changes in study participants’ attitudes. Four (36⋅4 %) studies^(21,22,32,33)^ reported a significant change in attitude after the intervention, while seven (63⋅6 %) studies^(16,18,24,26–28)^ reported no significant change. Schlair *et al.*^(22)^ observed medical students developing the attitude that patients are more likely to lose weight if they are counselled by physicians about weight and obesity management immediately following the intervention compared with baseline. Two studies found statistically significant increases in the odds of medical students reporting that nutrition counselling should be made a routine practice in medical care^(32,33)^, as well as the belief that physician counselling could increase the odds of patients wanting to improve their diet^(33)^. A study conducted in the United States with 718 first- to fourth-year medical students discovered a significant reduction in their perception of obese people having low intelligence and sexual attractiveness after intervention when compared with baseline^(21)^. Another study found that after intervention, participants’ attitudes towards reading and understanding nutrition labels for healthy food choices improved^(28)^. The seven studies that did not report a significant change in attitude post-intervention reported positive attitudes towards nutrition at baseline that were maintained post-intervention. Wood *et al.*^(18)^ discovered that at baseline, participants were both highly motivated (mean = 8⋅2 points) and excited (mean = 8⋅2 points) to counsel patients on healthy lifestyle choices, which was sustained post-intervention. Similarly, at the outset of three other studies^(24,26,34)^, almost all students believed that talking to patients about healthy lifestyles was important, and some believed that nutritional counselling could lead to improved dietary behaviours.

### Self-efficacy in nutrition care

More than half of the studies (*n* 13, 56⋅5 %) examined study participants’ self-efficacy^(12–14,16–18,22–24,26,28,34,35)^. Immediately following the intervention, eleven of the included studies found a significant improvement in participants’ self-efficacy to provide nutrition care^(12–14,16–18,22,24,28,34,35)^. The aspects that were improved were increased efficacy in using the plate dietary method, making appropriate referrals, and confidence in providing dietary counselling in the treatment of people with obesity, elevated LDL-cholesterol and type 2 diabetes. Following a qualitative approach, Rothmans *et al.* reported that some of the students who participated in their intervention commented that, unlike before, they are now confident in their dietary care prowess and thus willing to provide nutrition care^(12)^. In terms of follow-up assessments, Berz *et al.* reported a 1-month follow-up self-efficacy assessment, and while the score decreased from 4⋅1 to 3⋅9, it remained higher than the baseline score of 3⋅3.

The two studies that did not report significant improvements in self-efficacy either reported a significant decrease in participants’ confidence in providing nutrition care and health-related counselling to family/friends^(23)^ or reported a non-significant increase in confidence in discussing lifestyle issues with patients^(26)^. Crowley *et al.* explained that students’ confidence dropped as a result of the intervention's emphasis on the physiological aspects of nutrition rather than behaviour counselling^(23)^.

### Dietary and lifestyle habits of medical students and residents participating in NEIs

Nine (39⋅1 %) of the included studies^(12,18,22,23,26,28,32,33,35)^) looked at the impact of nutrition education on the dietary and lifestyle habits of medical students and residents. Except for one^(26)^, all of these studies (*n* 8, 89 %) improved participants’ dietary habits post-intervention^(12,18,22,23,28,32,33,35)^. Some of the improvements included increased fruit consumption, increased consumption of homemade food over restaurant and pre-prepared meals, avoidance of fatty foods, increased frequency of wholegrain food intake and decreased consumption of processed meat, avoiding the consumption of high sugar and fat foods, decreased odds of daily soft drink consumption and decreased odds of believing that healthy eating is expensive and time-consuming. The remaining study, Nawaz *et al.*, reported an insignificant change in the eating habits of their study participants with a statistically significant improvement in their ability to manage their own stress^(26)^.

### Preparedness of medical students and residents to provide nutrition care

Three studies looked into how prepared participants were to provide nutrition care^(16,18,28)^. In the study by Woods *et al*., participants’ self-rated level of preparedness increased significantly both immediately post-intervention (coefficient = 2⋅8 points; 95 % CI 1⋅6, 4⋅0 points; *P* = 0⋅001) and 2 months later (M: 2⋅2, 95 % CI 1⋅0, 3⋅4, *P* = 0⋅002). Mota *et al.* investigated the proportion of medical students who believed they could provide dietary counselling as a preventive measure before and after taking a nutrition and metabolism course during a semester. The authors discovered that the percentage of students who agreed they felt capable of providing dietary counselling increased significantly from 31⋅6 % at baseline to 73⋅3 % immediately post-intervention^(28)^. Lang *et al.*^(16)^ examined preventive medicine residents’ self-rated level of feeling as a competent cook and discovered that 50 % perceived themselves as competent cooks, which increased significantly to 67 % immediately post-intervention.

### Participants’ assessments of NEIs

Bert *et al.* and Rothmans *et al.* reported in their evaluation of participants’ experiences with the interventions that participants identified the need for the intervention to be extended to all medical students, the nutrition education programme being their best experience over their 4 years of medical education, and rating of the programme as their most preferred course since coming to medical school^(12,14)^.

## Discussion

We conducted a scoping review of the literature and summarised the effects of NEIs on the nutrition-related knowledge, attitude, self-efficacy, dietary and lifestyle habits, and preparedness to provide nutrition care among medical students and residents. We examined twenty-three studies that reported various interventions to improve the nutrition education experiences of medical students and residents, with significant improvements in the outcome variables reported. NEIs, in particular, were effective in improving participants’ nutrition-related knowledge, attitudes and self-efficacy following intervention. Furthermore, as reported in a number of the included studies, the personal dietary and lifestyle habits of medical students and residents improved post-intervention. Finally, the NEIs improved medical students’ and residents’ readiness to provide effective nutrition care in the future. These findings show that by implementing appropriate NEIs, it is possible to address the problem of inadequate nutrition education in the medical curriculum.

The twenty-three included studies in this review demonstrate the growing recognition of the need to improve medical students’ nutrition education experiences. Furthermore, critical stakeholders such as medical educators and researchers are showing an interest in finding solutions to the long-standing issue of inadequate nutrition education in the medical curriculum. It is also worth noting that almost all the interventions were designed to improve important competency outcomes such as nutrition-related knowledge, self-efficacy and attitudes, and personal dietary habits, all of which are required competencies for medical students and residents to provide effective nutrition care.

Nutrition-related knowledge is an important component of providing effective nutrition care, but it is not the only factor or attribute required. It is thus commonplace to find that twenty-one of the included studies designed studies aimed at improving participants’ nutrition-related knowledge, with more than 70 % (*n* 18) of those studies reporting significant improvements. This finding is consistent with the findings of the realist review reported by Mogre *et al.*^(6)^, in which the majority of the included studies reported improvements in participants’ nutrition-related knowledge. Similarly, Sunguya *et al.*^(7)^ discovered in their systematic review that eighteen of the twenty-five included studies reported significant post-nutrition training improvements in health workers’ nutrition knowledge. Despite these gains in knowledge, it is important to note that increased nutrition knowledge alone is insufficient to promote effective nutrition care delivery. This is supported by the findings of Mogre *et al.*^(6)^ who found that interventions aimed solely at increasing knowledge were less likely to change nutrition practice behaviour.

Another significant finding of this scoping review was that the included studies demonstrated that medical students and residents had a positive attitude towards nutrition education and saw nutrition care as an important responsibility prior to intervention. The majority of the reviewed studies did not report significant changes in participants’ attitudes towards nutrition care because the authors of the included studies reported that the participants already had positive and favourable attitudes towards nutrition care at the baseline measurements. It is commendable that the NEIs maintained the participants’ positive attitudes, as this is an important determinant of doctors’ ability to provide effective nutrition care^(3,6)^.

Self-efficacy is an important predictor of nutrition care, so it is not surprising that some of the interventions were designed to improve participants’ nutrition self-efficacy. Previous reviews also discovered a substantial number of included studies that designed interventions to improve this phenomenon or conducting cross-sectional surveys to investigate this phenomenon^(3,6)^. Eleven of the thirteen studies that looked into this phenomenon found that participants’ nutrition self-efficacy improved significantly after the intervention. This is generally laudable, given that increased self-efficacy is likely to result in increased nutrition care provision. Mogre *et al.*^(6)^ reported in their realist review of identifying the mechanisms that demonstrate why some NEIs succeed in improving nutrition care practice that interventions were more likely to result in improving nutrition care practice if they reportedly improved the participants’ nutrition self-efficacy.

In a realist review, Mogre *et al.*^(6)^ discovered that doctors will feel more at ease and more likely to provide nutrition care if they themselves practice healthy dietary and lifestyle habits. Several studies included in this review also investigated this concept. Following this concept, nine of the included studies designed interventions to improve the participants’ personal dietary and lifestyle habits, with the majority of them reporting post-intervention improvements in this phenomenon. These findings highlight the importance of improving nutrition education, as it will not only improve the competencies of medical students and residents, but will also help them change their dietary habits, lowering their risk of developing non-communicable diseases.

We also discovered that studies with post-intervention follow-up assessments/measurements found a decrease in knowledge and self-efficacy. This finding is concerning because the majority of the interventions included in this scoping review were single required courses delivered as a one-time event primarily during the preclinical level (some as early as the first and second years of medical school) with no opportunity for reinforcement^(22)^. Even though these interventions reported significant changes of the outcomes, the sustainability of these acquired competencies till practice will be difficult in the absence of opportunities for reinforcement. Furthermore, as demonstrated in a previous review, the majority of these interventions focused on increasing participants’ knowledge, which does not always translate into practice^(6)^. Nutrition self-efficacy and nutrition care expectations, as noted by Schlair *et al*.^(22)^ may be difficult to address in the early preclinical years due to a lack of clinical exposure. Integration of nutrition content throughout the entire medical programme, with opportunities for clinical exposure and reinforcement, may be more effective and should be recommended.

Except for one, all the included studies used multiple teaching and learning strategies to achieve the interventions’ intended outcomes. This is consistent with the findings of the systematic reviews published by Mogre *et al.*^(6)^ and Sunguya^(7)^. The use of multiple teaching and learning strategies is most likely informed by the fact that the studies had multiple outcomes that necessitated the use of various approaches. There is also evidence that interventions that used multiple approaches to meet participants’ educational needs were more likely to report significant changes^(6)^. In the findings of a previous review^(6)^, the use of multiple, non-traditional teaching and learning strategies was found to improve nutrition care competencies as well as nutrition practice behaviour^(4)^. Simulated patient cases, group work, role-plays, hands-on demonstrations, group practice, panel discussions, case-based learning, problem-based learning tutorials, computer or web-based cases, student-led debates, self-assessment exercises, interactive lectures and clinical case presentations were some of the non-traditional approaches used in the included studies in this scoping review.

Culinary medicine is a new and innovative approach to nutrition education that has gained popularity in recent years. Six of the included studies used this teaching model, which resulted in significant improvements in students’ confidence in nutrition counselling, home meal preparation, nutrition-related knowledge, personal cooking skills and dietary behaviour^(12,16,18)^. Its success may be due to its ability to provide hands-on, experiential learning opportunities that encourage active participation and strong interprofessional collaboration^(36,37)^. Thus, culinary medicine adds to the toolbox of approaches that could be used to improve the nutrition education experience of medical students and residents.

The strength of this review lies in the use of a comprehensive and extensive search strategy to identify relevant nutrition education interventional studies to provide an evidence account of the impact of the interventions. Despite its strengths, there are some significant limitations to consider. The majority of the included studies provided a limited description of the processes of intervention implementation relating to the ‘what’, ‘why’ and ‘how’ outcomes of the interventions were achieved, making it difficult for successful interventions to be repeated and adopted in other contexts and settings. Furthermore, because we only included articles in English, articles in other languages that may contain very rich information relevant to the purpose of this study may be overlooked. Only a few of the studies included studies had follow-up surveys, which provided limited information about the long-term impact of nutrition education on participants’ competencies, dietary and lifestyle habits.

## Conclusion

We found a diverse range of NEIs that used a variety of teaching and learning approaches, as well as a relatively large number of studies that could support a systematic review and meta-analysis, while taking into account the various designs and instruments, and advocating for nutrition education researchers to develop uniform instruments for assessing intervention outcomes. Despite this, the interventions showed promise in improving nutrition-related knowledge, self-efficacy, attitudes and preparedness to provide nutrition care among medical students and residents. The interventions also demonstrated their potential for improving the personal dietary and lifestyle habits of medical students and residents, which is required to potentially provide them with confidence to support individuals in adopting healthy dietary practices. Future NEIs should aim to follow-up with doctors beyond medical school, in order to assess how they use the acquired nutrition care competencies and skills in patient care to improve clinical outcomes.

## References

[1] Murray CJ (2019) Health effects of dietary risks in 195 countries, 1990–2017: a systematic analysis for the Global Burden of Disease Study. Lancet 393, 1958–1972. 30954305 10.1016/S0140-6736(19)30041-8 PMC6899507

[2] Rutledge T, Groesz L, Linke S, (2011) Behavioural weight management for the primary careprovider. Obes Rev 12, e290–e297. 21348915 10.1111/j.1467-789X.2010.00818.x

[3] Crowley J, Ball L & Hiddink GJ (2019) Nutrition in medical education: a systematic review. Lancet Planet Health 3, e379–e389. 31538623 10.1016/S2542-5196(19)30171-8

[4] Mogre V, Stevens FC, Aryee PA, (2018) Why nutrition education is inadequate in the medical curriculum: a qualitative study of students’ perspectives on barriers and strategies. BMC Med Educ 18, 1–11. 29433505 10.1186/s12909-018-1130-5 PMC5809975

[5] Hark LA, Deen DD & Morrison G (2015) Learner-directed nutrition content for medical schools to meet LCME standards. J Biomed Educ 2015, 469351.

[6] Mogre V, Scherpbier AJ, Stevens F, (2016) Realist synthesis of educational interventions to improve nutrition care competencies and delivery by doctors and other healthcare professionals. BMJ Open 6, e010084 10.1136/bmjopen-2015-010084 PMC5093684 27797977

[7] Sunguya BF, Poudel KC, Mlunde LB, (2013) Effectiveness of nutrition training of health workers toward improving caregivers’ feeding practices for children aged six months to two years: a systematic review. Nutr J. 12, 66. 23688174 10.1186/1475-2891-12-66 PMC3668136

[8] Arksey H & O'Malley L (2005) Scoping studies: towards a methodological framework. Int J Soc Res Methodol 8, 19–32.

[9] Levac D, Colquhoun H & O'Brien KK (2010) Scoping studies: advancing the methodology. Implement Sci 5, 1–9. 20854677 10.1186/1748-5908-5-69 PMC2954944

[10] Tricco AC, Lillie E, Zarin W, (2018) PRISMA extension for scoping reviews (PRISMA-ScR): checklist and explanation. Ann Intern Med 169, 467–473. 30178033 10.7326/M18-0850

[11] Steinert Y, Mann K, Centeno A, (2006) A systematic review of faculty development initiatives designed to improve teaching effectiveness in medical education: BEME Guide No. 8. Med Teacher 28, 497–526. 10.1080/01421590600902976 17074699

[12] Rothman JM, Bilici N, Mergler B, (2020) A culinary medicine elective for clinically experienced medical students: a pilot study. J Altern Complement Med (New York, NY) 26, 636–644. 10.1089/acm.2020.0063 32543207

[13] Caines L, Asiedu Y, Dugdale T, (2018) An interprofessional approach to teaching nutrition counseling to medical students. MedEdPORTAL 14, 10742. 30800942 10.15766/mep_2374-8265.10742 PMC6342344

[14] Berz J, Donovan K & Eyllon M (2020) An interprofessional nutrition education session for senior medical students on evidence-based diet patterns and practical nutrition tips. MedEdPORTAL 16, 10876. 32051854 10.15766/mep_2374-8265.10876 PMC7012311

[15] Rubin DJ & McDonnell ME (2010) Effect of a diabetes curriculum on internal medicine resident knowledge. Endocr Pract 16, 408–418. 20061294 10.4158/EP09275.OR

[16] Lang RD, Jennings MC, Lam C, (2019) Community culinary workshops as a nutrition curriculum in a preventive medicine residency program. MedEdPORTAL 15, 10859. 32051842 10.15766/mep_2374-8265.10859 PMC7010195

[17] Pang B, Memel Z, Diamant C, (2019) Culinary medicine and community partnership: hands-on culinary skills training to empower medical students to provide patient-centered nutrition education. Med Educ Online 24, 1630238. 10.1080/10872981.2019.1630238 PMC6609327 31248353

[18] Wood NI, Gleit RD & Levine DL (2021) Culinary nutrition course equips future physicians to educate patients on a healthy diet: an interventional pilot study. BMC Med Educ 21, 1–11. 34001085 10.1186/s12909-021-02702-y PMC8127510

[19] Afaghi A, Haj Agha Mohamadi AA, Ziaee A, (2011) Effect of an integrated case-based nutrition curriculum on medical education at Qazvin University of Medical Sciences, Iran. Global J Health Sci 4, 112–117. 10.5539/gjhs.v4n1p112 PMC4777021 22980104

[20] Sjarif DR, Yuliarti K, Wahyuni LK, (2016) Effectiveness of a comprehensive integrated module using interactive lectures and workshops in understanding and knowledge retention about infant feeding practice in fifth year medical students: a quasi-experimental study. BMC Med Educ 16, 210. 27538528 10.1186/s12909-016-0705-2 PMC4991091

[21] Gayer GG, Weiss J & Clearfield M (2017) Fundamentals for an osteopathic obesity designed study: the effects of education on osteopathic medical students’ attitudes regarding obesity. J Am Osteopath Assoc 117, 495–502. 28759091 10.7556/jaoa.2017.099

[22] Schlair S, Hanley K, Gillespie C, (2012) How medical students’ behaviors and attitudes affect the impact of a brief curriculum on nutrition counseling. J Nutr Educ Behav 44, 653–657. 22421794 10.1016/j.jneb.2011.08.006

[23] Crowley J, Ball L, Leveritt MD, (2014) Impact of an undergraduate course on medical students’ self-perceived nutrition intake and self-efficacy to improve their health behaviours and counselling practices. J Prim Health Care 6, 101–107. 24892126

[24] Coppoolse HL, Seidell JC & Dijkstra SC (2020) Impact of nutrition education on nutritional knowledge and intentions towards nutritional counselling in Dutch medical students: an intervention study. BMJ Open 10, e034377. 10.1136/bmjopen-2019-034377 PMC7200028 32284389

[25] Spear S, Sim V, Moore FA, (2013) Just say no to intensive care unit starvation: a nutrition education program for surgery residents. Nutr Clin Pract 28, 387–391. 23459609 10.1177/0884533613477136

[26] Nawaz H, Petraro PV, Via C, (2016) Lifestyle medicine curriculum for a preventive medicine residency program: implementation and outcomes. Med Educ Online 21, 1–7. 10.3402/meo.v21.29339 PMC4978856 27507540

[27] Walsh CO, Ziniel SI, Delichatsios HK, (2011) Nutrition attitudes and knowledge in medical students after completion of an integrated nutrition curriculum compared to a dedicated nutrition curriculum: a quasi-experimental study. BMC Med Educ 11, 58. 21835040 10.1186/1472-6920-11-58 PMC3173384

[28] Mota IB, Castelo I, Morais J, (2020) Nutrition education in Portuguese medical students: impact on the attitudes and knowledge. Acta Med Port 33, 246–251. 32238238 10.20344/amp.11817

[29] Magallanes E, Sen A, Siler M, (2021) Nutrition from the kitchen: culinary medicine impacts students’ counseling confidence. BMC Med Educ 21, 88. 33541352 10.1186/s12909-021-02512-2 PMC7863372

[30] Desalvo DJ, Greenberg LW, Henderson CL, (2012) A learner-centered diabetes management curriculum: reducing resident errors on an inpatient diabetes pathway. Diabetes Care 35, 2188–2193. 22875227 10.2337/dc12-0450 PMC3476896

[31] Lewis KO, Frank GR, Nagel R, (2014) Pediatric trainees’ engagement in the online nutrition curriculum: preliminary results. BMC Med Educ 14, 190. 25223502 10.1186/1472-6920-14-190 PMC4179838

[32] Monlezun DJ, Dart L, Vanbeber A, (2018) Machine learning-augmented propensity score-adjusted multilevel mixed effects panel analysis of hands-on cooking and nutrition education versus traditional curriculum for medical students as preventive cardiology: multisite cohort study of 3,248 trainees over 5 years. BioMed Res Int 2018, 5051289. 10.1155/2018/5051289 PMC5925138 29850526

[33] Monlezun DJ, Leong B, Joo E, (2015) Novel longitudinal and propensity score matched analysis of hands-on cooking and nutrition education versus traditional clinical education among 627 medical students. Adv Prev Med 2015, 656780. 10.1155/2015/656780 PMC4578831 26435851

[34] Frates EP, Xiao RC, Simeon K, (2016) Increasing knowledge and confidence in behavioral change: a pilot study. Prim Care Companion CNS Disord 18, 27409. 10.4088/PCC.16m01962 27922227

[35] Magallanes E, Sen A, Siler M, (2021) Nutrition from the kitchen: culinary medicine impacts students’ counseling confidence. BMC Med Educ 21, 1–7. 33541352 10.1186/s12909-021-02512-2 PMC7863372

[36] Aspry KE, Van Horn L, Carson JAS, (2018) Medical nutrition education, training, and competencies to advance guideline-based diet counseling by physicians: a science advisory from the American Heart Association. Circulation 137, e821–e841. 29712711 10.1161/CIR.0000000000000563

[37] Crawford AL & Aspry KE (2016) Teaching doctors-in-training about nutrition: where are we going in 2016? Rhode Island Med J 99, 23–25. 26929967

